# Much beyond Mantel: Bringing Procrustes Association Metric to the Plant and Soil Ecologist’s Toolbox

**DOI:** 10.1371/journal.pone.0101238

**Published:** 2014-06-27

**Authors:** Francy Junio Gonçalves Lisboa, Pedro R. Peres-Neto, Guilherme Montandon Chaer, Ederson da Conceição Jesus, Ruth Joy Mitchell, Stephen James Chapman, Ricardo Luis Louro Berbara

**Affiliations:** 1 Soil Science Department, Agronomy Institute, Federal Rural University of Rio de Janeiro, Seropédica-RJ, Brazil; 2 Canada Research Chair in Spatial Modelling and Biodiversity; Université du Québec à Montréal, Département des sciences biologiques, Québec, Canada; 3 Embrapa Agrobiologia, Seropédica-RJ, Brazil; 4 The James Hutton Institute, Craigiebuckler, Aberdeen, United Kingdom; University of Westminster, United Kingdom

## Abstract

The correlation of multivariate data is a common task in investigations of soil biology and in ecology in general. Procrustes analysis and the Mantel test are two approaches that often meet this objective and are considered analogous in many situations especially when used as a statistical test to assess the statistical significance between multivariate data tables. Here we call the attention of ecologists to the advantages of a less familiar application of the Procrustean framework, namely the Procrustean association metric (a vector of Procrustean residuals). These residuals represent differences in fit between multivariate data tables regarding homologous observations (e.g., sampling sites) that can be used to estimate local levels of association (e.g., some groups of sites are more similar in their association between biotic and environmental features than other groups of sites). Given that in the Mantel framework, multivariate information is translated into a pairwise distance matrix, we lose the ability to contrast homologous data points across dimensions and data matrices after their fit. In this paper, we attempt to familiarize ecologists with the benefits of using these Procrustean residual differences to further gain insights about the processes underlying the association among multivariate data tables using real and hypothetical examples.

## Introduction

In multidimensional data analysis, ecologists often encounter situations where they need to choose between two or more numerical approaches that are able to tackle the same question of interest. The preference between approaches is based, among other factors, on the familiarity of the user with the method, which in turn depends on the time a particular method has been available in statistical packages and the ease in implementing and interpreting its results. Another relevant factor to consider is “literature–induced use” in which renowned research groups involved in the development, improvement and generation of statistical ecological approaches have a strong influence on the types of statistical approaches other ecologists use.

**Figure 1:**
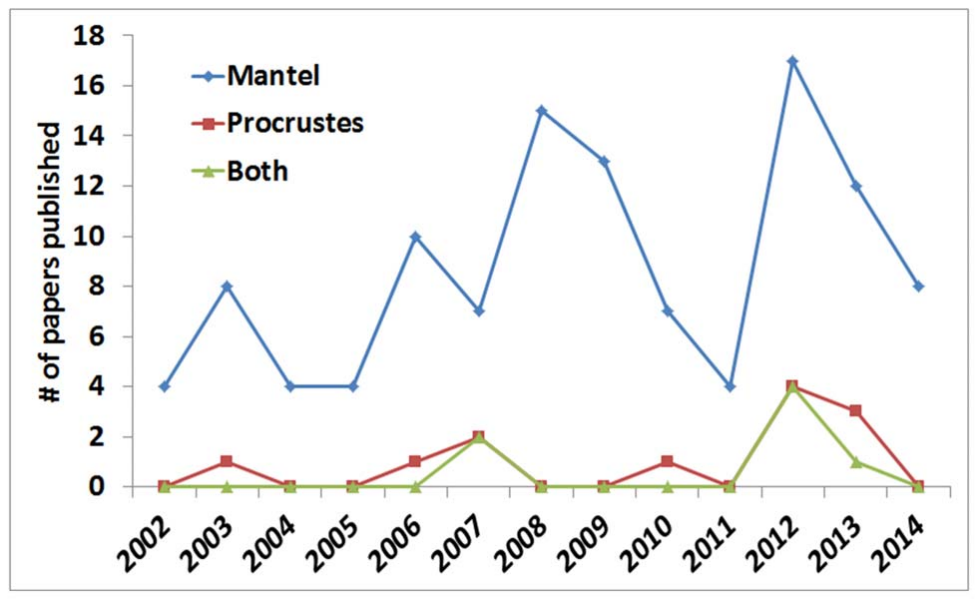
Papers published using Mantel and Procrustes for relating data matrices from soil or plant studies in the ten years since [3] stated the advantages of Procrustes over the Mantel approach. Data obtained using Thompson Reuters database (May, 12, 2014). We searched for papers using uniquely the Mantel approach, uniquely the Procrustes approach and papers using both approaches. The search was based on Procrust* (Procrustean or Procrustes) and PROTEST.

Determining the strength of the relationships between multivariate datasets is a routine analysis when trying to understand the environmental factors driving the composition and structure of ecological communities. Two approaches, the Mantel test [1] and Procrustes analysis [2], though considered analogous by the literature in the questions they can tackle [3], have not been used to the same extent. Despite the advantages of Procrustes analysis over the Mantel test [3] regarding greater statistical power in detecting significant relationships (i.e., lower type II errors) and the possibility of analyzing further the patterns of association between multivariate matrices (visually and by further statistical analyses), the Procrustean approach remains relatively unused in tackling questions regarding the relationships between data matrices involving plant and soil information or between soil matrices (Fig. 1).

The Mantel test and the Procrustes approach can be both used in many similar situations where the aim is to assess how multivariate data matrices are associated (correlated), though for unknown reasons they have been used in quite different ways in the ecological literature. For example, while the Mantel test has often been applied when testing the relationship between above and below ground data matrices [4], [5], [6], [7], [8], [9], [10], [11], [12], Procrustes analysis has predominantly been used to contrast the results of different ecological ordinations on the same data [13], [14], [15], [16], to compare fingerprinting tools for assessing microbial communities [17], [18], [19] and for deciding between methodological choices [20], [21]. Indeed the Procrustean framework has been rarely used to make inferences about plant and soil relationships [22], [23], [24], [25], [26] and other types of ecological associations between data sets. However there are instances in which the Procrustean and Mantel tests cannot be used interchangeably. Unlike Mantel, the Procrustean approach can be used to compare multiple data matrices. However, when ecologists are interested in correlating distance (or similarity) matrices, rather than testing the association among data matrices in their raw form (i.e., not transformed by the property of distance measures), Mantel, rather than Procrustes, is more appropriate. One particular case is the distance-decay of similarity in ecological communities [27] in which one is interested in testing the hypothesis that the similarity in community composition decreases in relation to linear (or log transformed) geographic distance between communities. The differences between raw-based and distance-based approaches have been discussed extensively elsewhere [28], [29].

Despite the relative merits of the Procrustean framework over the Mantel test shown by the relatively well-cited paper by Peres-Neto and Jackson [3], its potential has not yet been tapped. Perhaps the reason for Procrustes analysis not being as popular as the Mantel test among ecologists is the lack of a paper showing that in many situations traditionally investigated by Mantel, the Procrustean analysis can be equally well used. Here, we attempt to familiarize ecologists with the use of Procrustes analysis by using real and hypothetical examples where the Mantel test tends to be preferred. Most importantly, we highlight little explored limits of Procrustes by using its residual vector of association between data tables, hereafter referred as to PAM, in three common statistical approaches: multivariate ordination, variation partitioning and ANOVA.

## Procrustes analysis: a foundation for soil and plant ecologists

In ancient Greek mythology there was a character named Procrustes who was a resident of Eleusis Mountain, a known travelers’ route. As a “good” host, Procrustes always invited travelers to spend the night at his home; more specifically, he invited them to lie down on his iron bed, which was tailored to fit Procrustes’ own body. The guests who did not fit the dimensions of his bed either had their limbs cut off or were stretched until their dimensions approached those of Procrustes’s bed. Ironically, none of the guests ever fitted the iron bed because Procrustes secretly had two beds of different sizes [30]. One can easily make a parallel here with ecological data in which data from different sources will almost never easily compare or fit to one another.

Procrustes analysis is based on the search for the best fit between two data tables, hereafter referred to as matrices, where one is kept fixed (“Procrustes’ bed” or target matrix), while the other (“Procrustes’ guest” or rotated matrix) undergoes a series of transformations (translation, mirror reflection and rotation; [2]) to fit the fixed matrix. Although in this paper we concentrate on fitting two matrices, the extension of Procrustes analysis to multiple matrices is straightforward [3] in which the reference matrix can be either one of the original matrices or their averages (or medians). Hereafter, the target matrix (target) will be referred as to **X**, and the data matrix to be fitted as **Y**. **X** and **Y** are both *n*×*p* matrices, where *n* is the number of rows and *p* is the number of columns. The goal of the transformations in **Y** is to minimize the residual sum of squared differences between the corresponding *n* dimensions between **X** and **Y**; the sum of the squares of these residual differences is termed *m* ^2^ (Gower’s statistic), representing the optimal fit between the two data matrices, such that the higher the value of *m* ^2^, the weaker the relationship between the two data tables is. The significance of *m* ^2^ can be estimated through a permutation test (termed PROTEST after [31]; see [3] for further details).

## Procrustean association metric (PAM)

The least squares superimposition between the corresponding *n* observations of **X** and **Y** is one of the main advantages (in addition to the increased statistical power) of the Procrustean framework in contrast to the Mantel test. The Procrustes superimposition generates a (*n*×*p*) matrix of residuals that can be further used to contrast the differences between homologous observations (rows) across matrices in the form of a vector (PAM). Given that within the Mantel approach differences between observations across all dimensions are packed down into a single distance, it cannot be used to assess differences across observations across dimensions. Consistent small and large differences across homologous observations across matrices in regard to other factors of interest can further assist in understanding how **X** and **Y** are related. For example, we could use PAM to assess the degree of observation matching between a plant function trait matrix and a composition matrix and assess whether smaller or greater residual values are a function of the time elapsed since some disturbance event.

PAM is simply a vector of residual differences between the corresponding *n* observations. For example, assuming that an ecologist wants to correlate two matrices of data **X** and **Y**, both of which are formed by four rows (i.e. sites, plots, observational units), Procrustes analysis will generate four residual differences between the **X** and **Y** configurations. The compilation of these residual differences between homologous rows (observations) across dimensions in the form of a vector – PAM – represents a useful way to represent information on the relationship between two matrices and make it available for further statistical analysis, both parametric and non-parametric; this feature is not offered by the Mantel approach.

The use of the residual vector from Procrustes (PAM) has been quite restricted in the plant and soil ecological literature. To our knowledge the first study was by [32] who assessed the plant-pollinator interaction during three consecutive summers in the southeastern portion of California, USA. These authors employed the PAM to identify which pollinating species exhibited the greatest deviation between two consecutive years. Singh et al. [22] used the PAM in a study on soil microbiology to verify the effect of soil pH on the relationship between arbuscular mycorrhizal fungi (AMF) and plant assemblages. These authors employed the following strategy: 1) Procrustes analysis was applied between the matrices representing the AMF community and that representing the plant community; 2) after detecting a significant relationship (*m*_12_ = 0.28; P<0.001), these authors extracted the PAM and used it as a response in a simple regression analysis with the soil pH. No effect of pH on the association between the AMF and plant communities was detected, suggesting that neither the pH nor the identity of the plant species that composed the community affected the AMF community. Other applications can be certainly found (e.g., [24], [25], [26] [33]) but its flexibility and general usage remains largely unexplored.

## Constructing a practical roadmap for applying PAM

There are few studies in the ecological literature that have used PAM for analyzing relationships between plant and soil datasets. The lack of examples partially explains the low popularity of Procrustes analysis among plant and soil ecologists and ecologists in general as an alternative tool to the more traditional Mantel test. In order to make the possible uses of Procrustean residuals more familiar, we will introduce a number of examples in the form of schematic roadmaps for applying PAM in association with three common statistical approaches: ordination, regression analysis and ANOVA.

**Figure 2:**
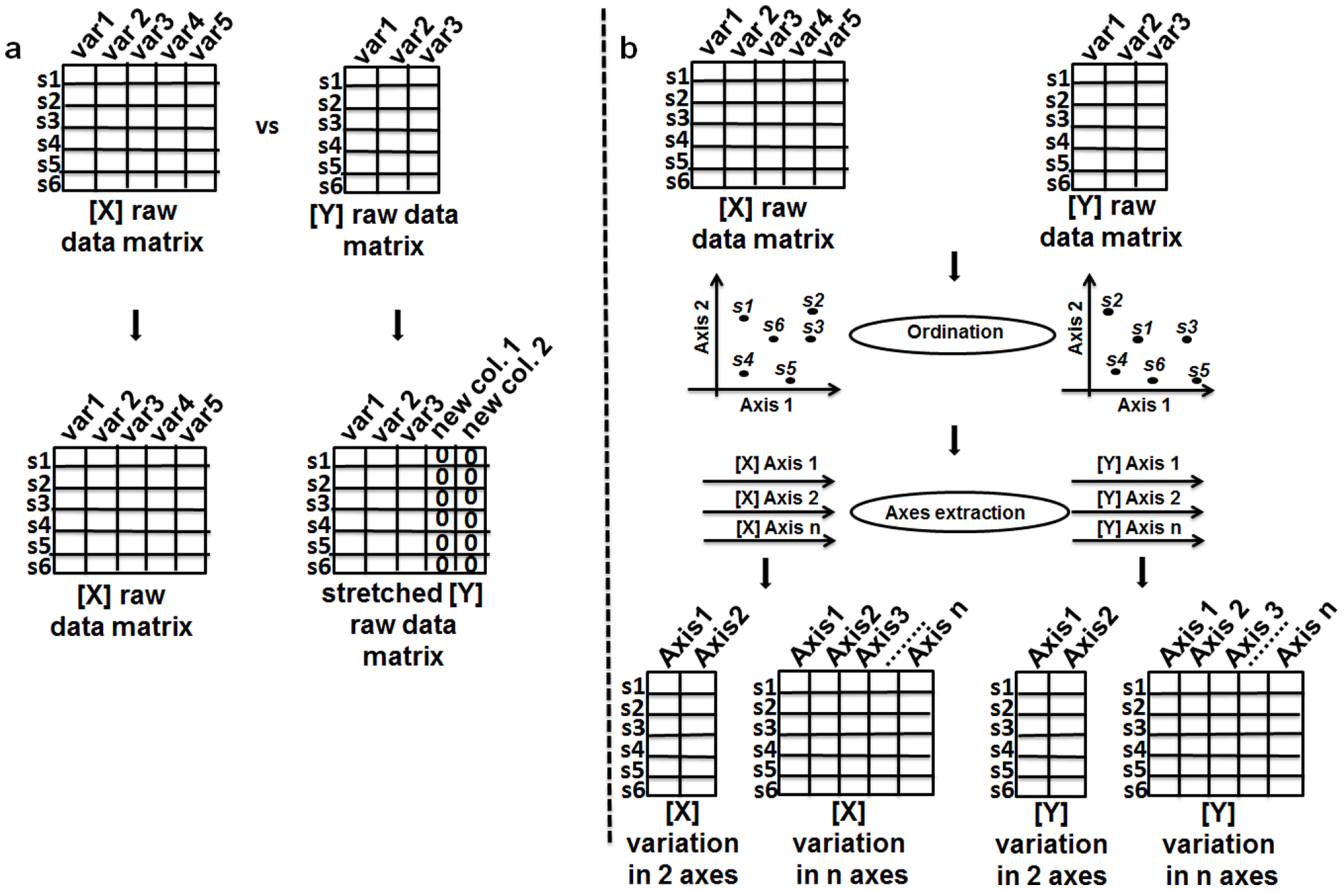
Roadmap for two alternative ways to reach the same dimensionality between matrices, and so relating it by Procrustes analysis. **a)** Addition of columns containing zeros to the **Y** raw data matrix for matching the **X** raw data matrix dimension; **b)** Application of ordination to raw data matrices to make matrices have equal dimensionality prior to Procrustes analysis.

Plant and soil ecologists must keep in mind that Procrustes analysis requires that the **X** and **Y** have the same number of rows and columns, though the last dimension is less restricting (see below). Given that the data for both matrices usually originate from the same sites, it is most common in ecology that only the number of columns (descriptors or variables) varies between the two matrices. Therefore, the question arises of how to make the number of columns equal across the two matrices, i.e., how to reduce them to the same dimensionality. Although Procrustes analysis can be performed between matrices having different number of dimensions (i.e., the fit is based on a singular value decomposition (svd) of X^T^Y, where X and Y are scaled prior to svd and T stands for matrix transpose), traditionally the matrix with the fewer number of columns (“missing columns”) is made equal in dimension to the larger matrix by adding columns of zeros in order to keep (Fig. 2a; [2]). Although there are some criticisms related to this practice and alternatives have been suggested [34], the addition of zero columns does not affect the distances between columns among observations and is a convenient device rather than a hurdle [35].

**Figure 3:**
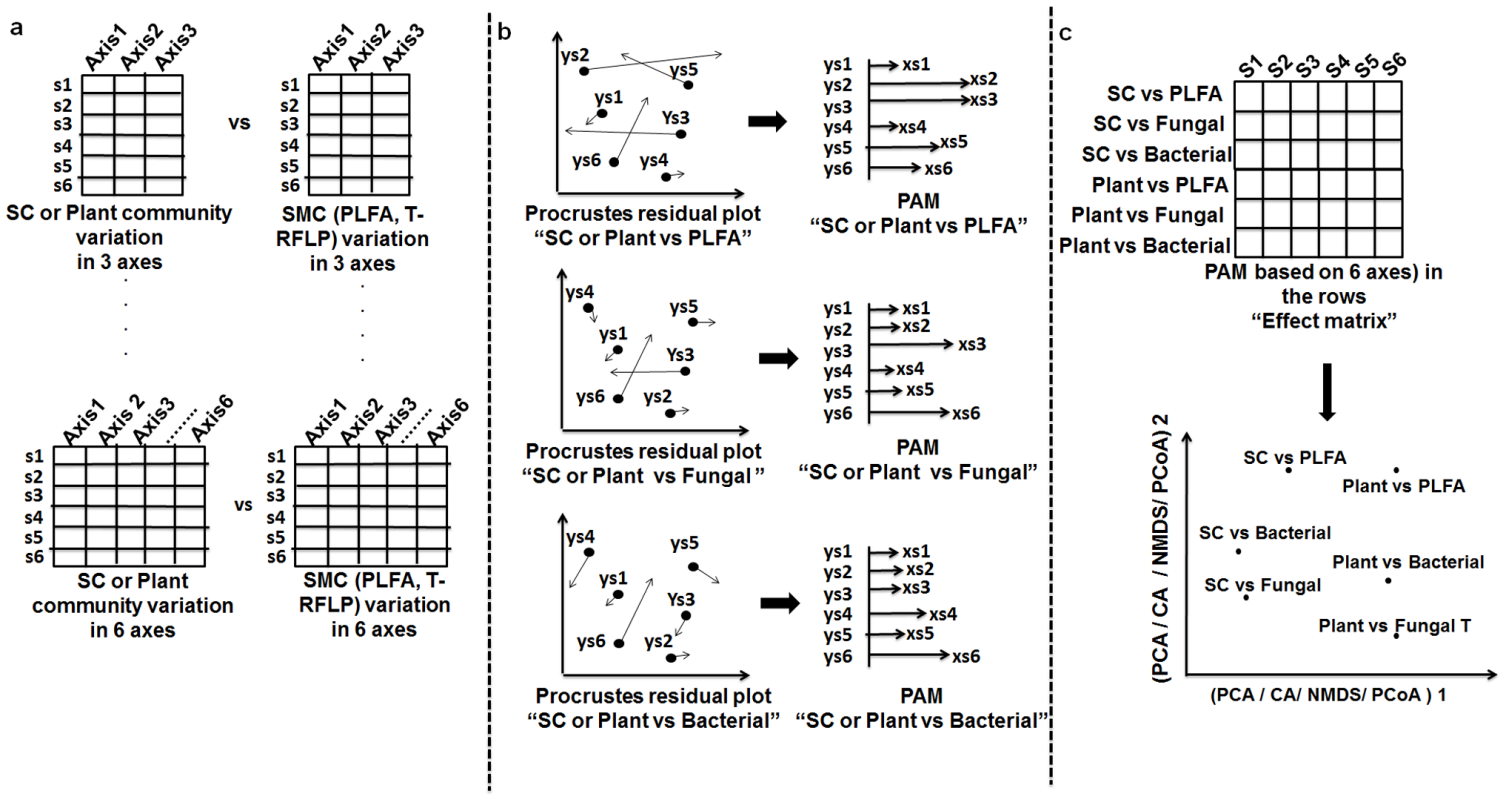
Roadmap for applying the Procrustes association metric (PAM) in the multivariate ordination context using data of [39]. **a)** Assembling matrices with different ordination axes, through Procrustes analysis, soil chemistry (SC) and plant community with soil microbial community (PLFA, and bacterial and fungal T-RFLP); **b)** Extraction of PAM from Procrustean relationships based on matrices with 6 ordination axes; **c)** Assembling of PAM based PCA matrices with 6 axes as rows in a single matrix (“effect matrix”), and using it in an ordination technique (e.g., PCA, PCoA, NMDS) to verify if the different effects diverge.

Another convenient way to make **X** and **Y** have the same number of columns is to represent most of the variation in their raw data by matrices formed by the same number of orthogonal axes (Fig. 2b; [3],[35, [36]), i.e., matrices formed by axes derived through ordination methods such as Principal Components Analysis (PCA), Non-metric dimensional scaling, Correspondence Analysis (CA), Principal Coordinate Analysis (PCoA), the choice being dependent on the nature of the data (continuous, presence-absence data, abundance data). Moreover, raw data matrices can be transformed prior to ordination (see [37] for different transformations and their characteristics) or alternatively have pairwise distance matrices calculated from the data matrices that are then orthogonolized via PCoA to extract ordination axes based on the chosen distance measure (e.g., Bray-Curtis, Jaccard, Sorensen, Gower).

Here, for simplicity, we use a PCA in all applications. In cases, where species data (presence/absence or abundance) was used, the data was Hellinger-transformed and PCAs were extracted on species correlation matrix calculated from the transformed data. The Hellinger transformation alleviates the issue of double-zeros in species data matrix transformed into correlation or Euclidean-distance pairwise matrices prior to PCA in which sites sharing no species in common can be found to be more similar than sites sharing a reduced number of species in common (e.g., the horse shoe effect in ordination plots).

The general strategy is as follows:

1. Subject the raw data matrices to an ordination method (here PCA but see above for other strategies);
2. After ordinating **X** and **Y**, use the same number of ordination axes for both matrices (Fig. 2b).

Given that the higher the number of ordination axes used, the higher is the amount of variation explained in **X** and **Y**, it would be interesting run the Procrustean analysis sequentially using matrices made up of an increasing number of ordination axes. It could help ecologists check the consistency of the relationship between **X** and **Y** based on different numbers of ordination axes, which will give more reliability to the results.

## The use of PAM in ecological ordination

The first form of PAM shown here is based on ordination methods. Ordination is the graphical representation of the variation of objects (sites), descriptors (species/environmental parameters) or both, in a reduced space formed by orthogonal axes [38].

To illustrate the use of Procrustes analysis associated with ordination we use data derived from Mitchell et al. [39]. This study aimed to compare the plant communities and soil chemistry in their ability to predict changes in the structure of the soil microbial community in three moorland areas established in Northern Scotland called Craggan, Kerrow and Tulchan. The plant community matrices from each area were based on the percent cover. Three matrices for the soil microbial community were obtained for each site: one based on the fatty acid profile of the soil (PLFA analysis), and the other two on the T-RFLP analysis of the communities of fungi and bacteria, respectively. The matrix representing the soil chemistry was based on the concentrations of Na, K, Ca, Mg, Fe, Al, P, total C, total N in addition to pH, loss on ignition and moisture.

There is some consensus that the variation in vegetation can act as a proxy for changes in the soil microbial community, either directly in the case of symbionts, for example, or indirectly via changes in soil chemistry itself. We use Procrustes analysis associated with ordination techniques to verify potential drivers of the soil microbial community and to determine if plant community and soil chemistry are equally related to the microbiological variation. The sequence of analysis was as follows:

1. Ordination analysis: All data matrices (community plant, soil chemistry and soil microbial communities) containing the three chronosequences were subjected to separate PCAs based on correlation matrix. The community plant was Hellinger-transformed prior to PCA. Then, the first six PCA ordination axes from each matrix were retained in order to assemble four PCA matrices representing the variation summarized in the first 3, 4, 5 and 6 PCA axes. Thus, four PCA matrices were obtained from each dataset: plant community, soil chemistry and soil microbial community (PLFA, bacterial and fungal T-RFLP) (Fig. 3a).
2. Procrustes analysis: The PCA matrices of plant community and soil chemistry were used to run Procrustean analyses with the PCA matrices of soil microbial community based on PLFA, and fungal and bacterial T-RFLP datasets.
3. PAM extraction: Since all Procrustean relationships based on PCA matrices with *n* axes were significant, for simplicity, only the PAM obtained from relationships of PCA matrices with 6 axes were used for subsequent analyses. Six PAMs were generated: PAM1 (soil chemistry on PLFA), PAM2 (soil chemistry on bacteria), PAM3 (soil chemistry on fungi), PAM4 (plant on PLFA), PAM5 (plant on bacteria), and PAM6 (plant on fungi) (Fig. 3c).
4. PAM ordination: The PAMs were assembled in a single matrix (“effect matrix”) with one PAM per row (Fig. 3c). Therefore, the effect matrix compiled the effects of plant community and soil chemistry on soil microbial community structure derived from the three methods. This effect matrix was submitted to PCA ordination to verify whether the plant community effect on soil microbial community structure differed from the effect of soil chemistry (Fig. 3c).

**Figure 4:**
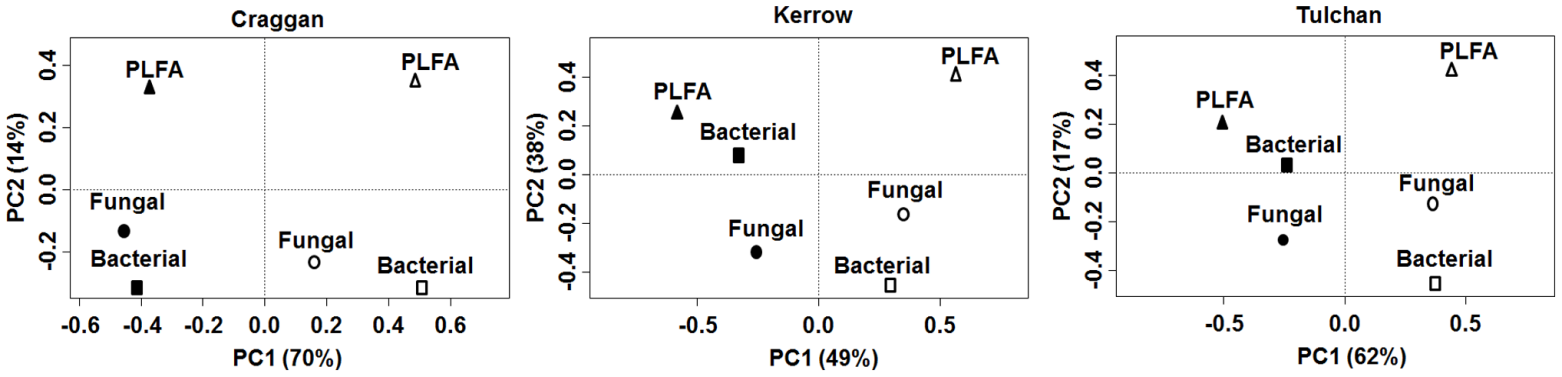
Results from PCA ordination of the Procrustes association metric matrix (“effect matrix”) gathering the interactions of soil chemistry and plant community with soil microbial matrices (PLFA, and bacterial and fungal T-RFLP). The filled symbols are the Procrustes relationships between soil chemistry and soil microbial matrices, and the open symbols between plant community and soil microbial matrices. Data from three chronosequences (Craggan, Kerrow and Tulchan) obtained by [39].

The results showed that for all chronosequences the plant effect on microbial structure was divergent in relation to the soil chemistry effect, as suggested by the separation along the axis of greatest variation (Fig. 4). Although we cannot apply a proper statistical significance test in one-table based ordination methods (PCA, NMDS, PCoA, etc), visual inferences can be made. For example the Craggan area exhibited a clear distortion between plant community and soil chemistry variation in terms of their effects on the soil microbial community structure depicted by PLFA, bacterial T-RFLP and fungal T-RFLP (Fig. 4a). Also in this area, the response of the microbial community based on PLFA was distant from the response based on molecular data (T-RFLP) (Fig. 4a),

We expected that the effects of soil chemistry on microbial structure were closer to the effect of plant community once the plant community is considered to be a direct and indirect driver for the biotic component of soil [39]. However, these results suggest that plant communities and soil chemistry are acting differently on the soil microbial community structure [24], [40]. They also suggest that the effects of soil chemical properties on the microbial communities may be weakly mediated by above ground alterations [24]. This example shows the usefulness of Procrustes analysis to raise additional evidence in plant and soil ecology studies. (See Text S1 containing the R code used for this example).

## The PAM and regression analysis

In regression analysis, ‘response’ and ‘predictor’ are common terms. In ecology, predictors can have different natures. Space, time, organic matter and moisture, among other factors, are some examples of predictors. On the other hand the microbial communities are often used as a response variable because they are considered better indicators of a given ecosystem.

**Figure 5:**
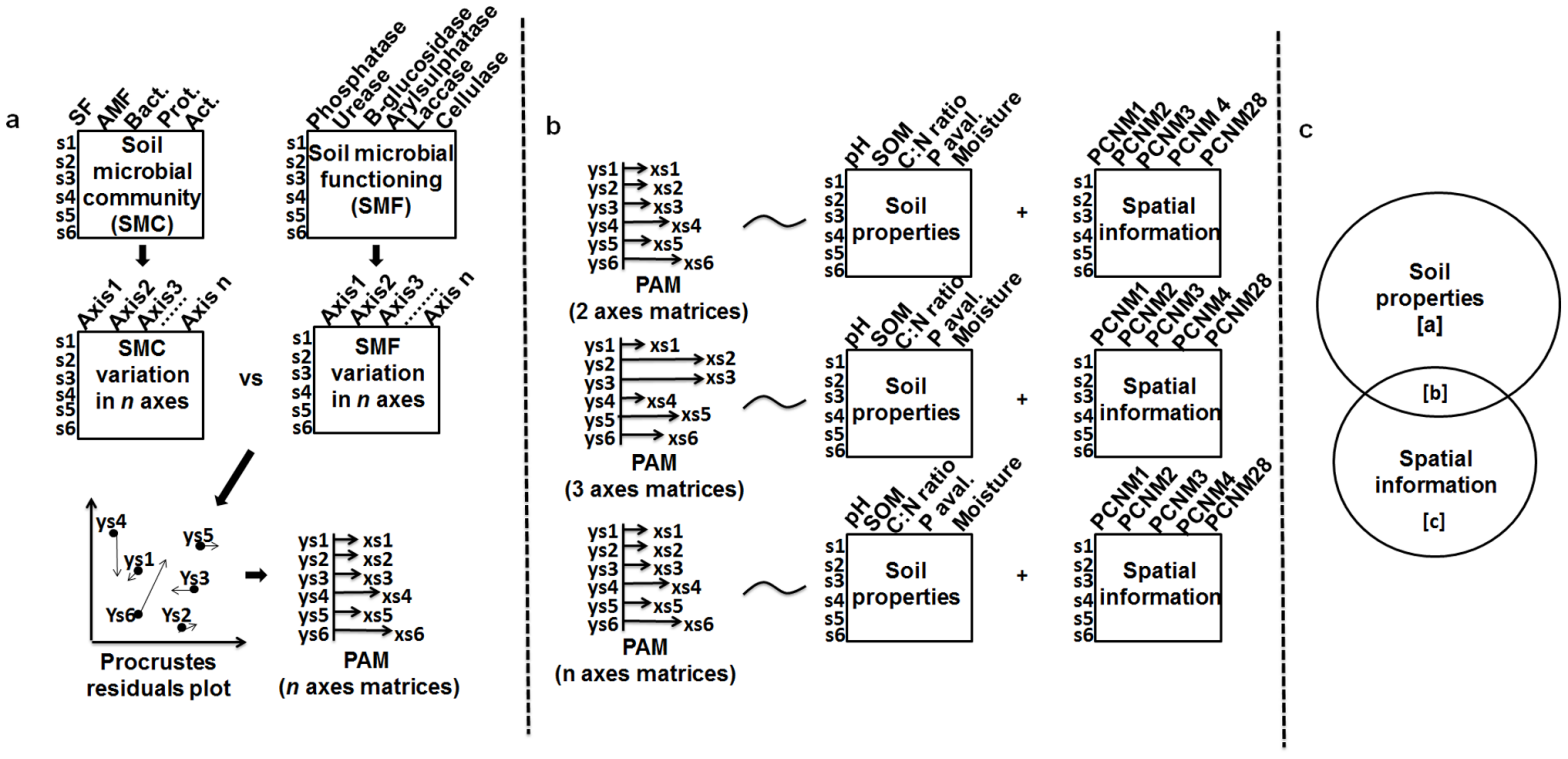
Roadmap for using Procrustes Association Metric (PAM) in a multiple regression analysis framework (variation partitioning). **a)** Soil microbial community (SMC) and soil microbial functioning (SMF) matrices are submitted to an ordination to reach the same dimensionality, and SMC and SMF matrices formed by 2, 3 and *n* axes related through Procrustes analysis in order to generate PAMs; **b)** PAMs generated were used as response variables in a variation partitioning to verify the individual contribution of soil properties and spatial information (PCNM eigenfunctions) on the SMC-SMF relationship; **c)** Venn diagram depicting the relative contribution of soil properties (niche processes **[a])** and unmeasured spatial factors (neutral processes **[c]**).

Some authors familiar with soil microbial ecology have been using the Mantel test to assess the individual contribution of deterministic and stochastic processes on the soil microbial structure variation [41], [42]. As an example of the utility of the Procrustes analysis in the context of variation partitioning we can take a hypothetical scenario with four datasets from a given area, corresponding to soil microbial community structure (PLFA), soil microbial functioning (enzyme activities), soil properties and spatial variation. Spatial variation can be represented, for example, by 100 sampling points generated from a 10 m×10 m transects. The matrix of geographical coordinates of the sampling points can be submitted to PCNM (principal coordinates neighbour matrix) analysis generating a matrix of spatial eigenfunctions termed PCNMs [34]. In this scenario, we can assume that the ecologist aims to assess the relative contributions of individual soil properties (deterministic processes) and spatial variation (stochastic event) on the relationship between microbial community structure and soil microbial functioning rather than on these components individually. To use the Procrustean association metric (PAM) in this context, one can use the following steps:

1. Ordinate the two matrices (i.e., the soil microbial community and soil microbial functioning) via PCA (the soil microbial community matrix was Hellinger-transformed) and select a similar number of ordination axes. The multivariate scores of the two matrices across the selected number of axes are subjected to a Procrustes analysis and a PAM was then calculated.
2. Use individual PAMs (based on 2, 3 or more PCA axes) as response variable and soil properties and spatial variation as independent (predictor) variables in a multiple regression framework (Fig. 5b).
3. Finally, the independent contributions of soil properties (independent of space) and unmeasured spatial process and/or factors (spatial variation independent of soil properties) to the microbial structure can be estimated via variation partitioning [43] and represented by a Venn diagram (Fig. 5c). (See Text S2 containing the R code for this example).

## The PAM and Analysis of Variance

Although regression and analysis of variance are ultimately the same analysis in which the response is either continuous (regression) or ascribed to factors (ANOVA), we provide examples for each of them in different sections given that often they are seen as distinct forms of analyses. Evaluation of the effects of land use on soil microbial communities has been a common case-study issue in soil ecology. Some of these studies have been carried out using the Mantel approach to assess how land use type effects soil microbial structure and functioning [44], [45]. However, Mantel does not yield a vector of structure – functioning relationship, that is, a continuous variable, able to be partitioned by categorical variables like land use types. In the following example we show how to use PAM to evaluate the effect of land use type on the relationship between microbial community structure and microbial function in the form of PAM.

**Figure 6:**
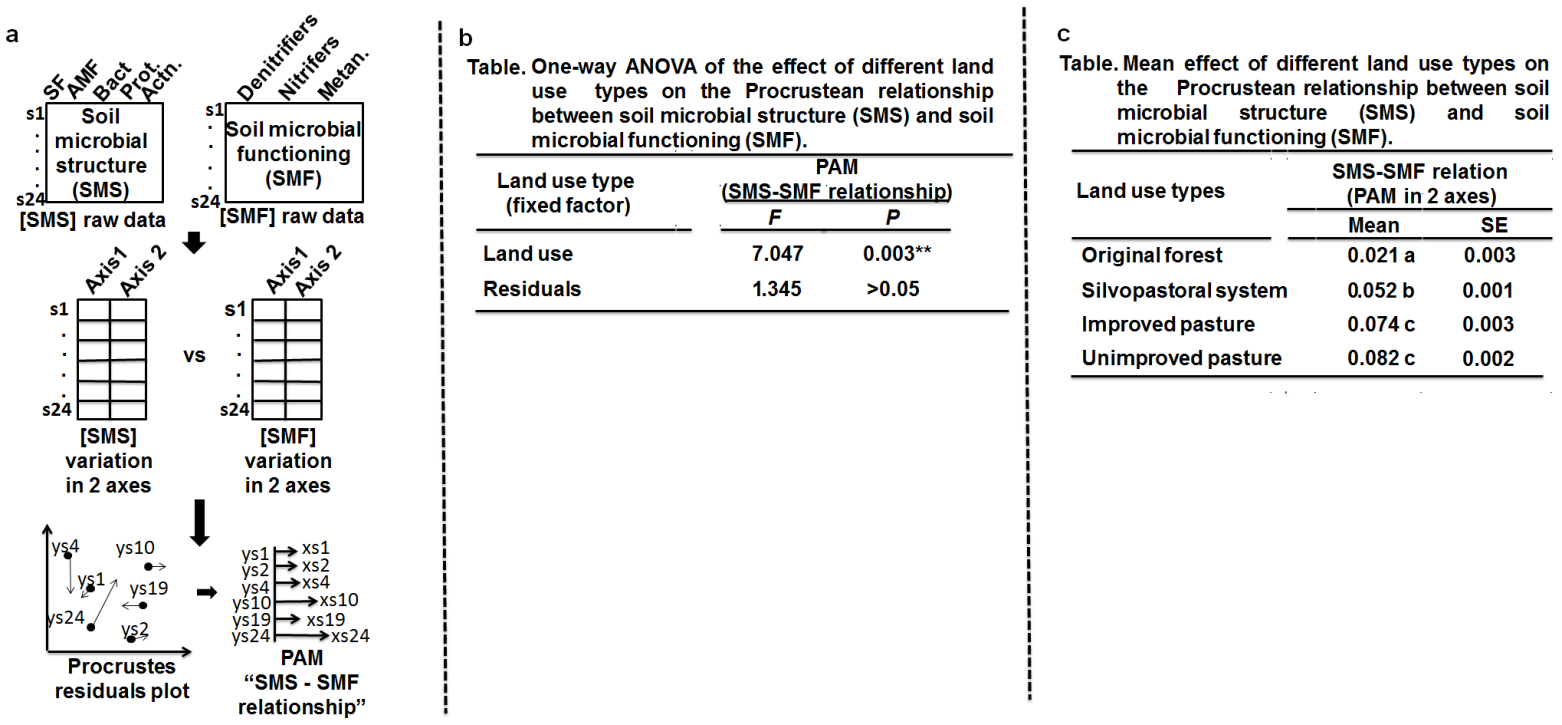
Roadmap for using Procrustes association metric (PAM) in an ANOVA context. **a)** PCA ordination of each SMC and SMF raw data matrices, and then Procrustes correlation from 2 axes-based PCA matrices in order to generate the PAM depicting the SMC-SMF relationship. **b)** Table showing results of a one-way ANOVA for using PAM as response and land use type as fixed factor. **c)** Multiple comparisons test (Tukey, 95%) for means of the Procrustean relationship between soil microbial structure and functioning (PAM in 2 axes) across land use types.

In a hypothetical scenario, a researcher is interested in studying whether four different land use types within the Amazon biome are affecting the relationship between microbial structure and microbial functioning. In each of the land uses (original forest fragment, silvipastoral system, improved pasture and unimproved pasture) six plots (10 m×10 m) were established and one composite soil sample (0–10 cm) collected per plot (Fig. 6a). The X dataset (soil microbial structure) was represented by PLFA data, and the Y dataset (microbial functioning) by the abundance of genes associated with microorganisms involved in greenhouse gas emission processes, such as nitrifiers, denitrifiers and methanotrophic organisms. The researcher’s hypothesis is that in the original forest (non-altered environment) there is a better matching between microbial structure and microbial function. Thus, in anthropogenically disturbed environments (silvipastoral system, improved pasture, and unimproved pasture) the change in microbial structure relative to the original (forest) is not followed by a change in the microbial functioning to the same magnitude. This hypothesis can be tested using an integration of Procrustes analysis and ANOVA through the following steps:

1. Reduce the datasets **X** (soil microbial structure) and **Y** (soil microbial functioning) to similar dimensions using PCA. Then, run the Procrustean analysis between the PCA matrix of the soil microbial community structure and the PCA matrix of soil microbial functioning and extract the PAM (Fig. 6a).
2. Run an ANOVA with land use type as fixed factor and the PAM as the response variable (Fig. 6b).
3. If the F value of ANOVA is significant, a means test can be performed to compare the mean PAMs of the land use types (Fig. 6c). (See Text S3 containing the R code for this example).

## Discussion

In this paper we have attempted to show the advantages of the Procrustean analysis over the Mantel test, in which the former can be used for gaining further information on underlying drivers of data table associations. In particular we have shown how the Procrustean association metric (PAM) constructed of the residuals of the vectors after the Procrustes analysis. We concentrated on showing how patterns of concordance between data matrices can be displayed and individual observations contrasted separately using the Procrustean framework, allowing further examination of the common and different association patterns among multiple data matrices. Given that in the Mantel framework, multivariate information is translated into a pairwise distance matrix, we lose the ability to contrast homologous data points across dimensions and data matrices. It is important to notice that it was not our goal to show the statistical advantages of Procrustes over Mantel as done by previous work [3]. Instead, we concentrated on generating different analytical schemes, especially for plant and soil ecologists, to incorporate Procrustes into their statistical toolbox.

What is unique about Procrustean framework? There are at least four characteristics of the approach not shared by others. First, because the approach is correlative rather than regressive, the number of observations (e.g., sites) in the matrices does not have to be greater than the number of columns as in common regression approaches such as RDA and CCA. Second, we can fit as many matrices as we have available; this latter issue is particularly restrictive under a regression approach given the limitation of number of rows versus number of columns. Moreover, all matrices are treated in equal footing as no matrix is treated as response or predictor. Third, the relationships within (only across) matrix columns do not affect the analysis. Fourth, residual values across observations and dimensions can be calculated and explored as shown here. These characteristics should not be necessarily seen as advantages per se over other methods but rather features that are unique and may be useful in many situations. There are certainly other tools that can be used to look at the associations between data sets. RDA and CCA are well-established tools in ecology and are based on regression (asymmetric) methods. Traditionally these approaches may have been thought to be more appropriate for analysis of the examples given in this paper, since they establish relations of cause and effect. However, because these analyses include a regression step, they are limited to situations where the number of rows (sites) in the environmental matrix **X** is higher than the number of columns (variables) [36], [46]. This is not a limitation in Procrustes analysis and moreover, it is not clear how residual variation among homologous observations across dimensions should be explored in the case of RDA and CCA.

At least two other symmetric approaches are similar to the Procrustean approach, namely Co-inertia analysis [47] and symmetric Co-correspondence analysis [48], a form of Co-inertia analysis in which a correspondence analysis is applied to two species matrices prior to the analysis. The main difference resides in the fact that fit is influenced by all variables pairs in Co-inertia analysis (within and between matrices), whereas fit is influenced only by variation between matrices in Procrustean. Co-inertia is always based on ordination within data matrices, whereas in Procrustes either the raw data or their ordination axes can be used. Co-inertia can also take into account row (e.g., sites) and column (e.g., species) weights in the analysis, though the standardization and fit processes in Procrustean analysis could also take these into account [36]. Co-inertia and Procrustean analysis are certainly related in the sense that they both treat matrices as symmetrical during the fitting process, though more studies are necessary to assess in which conditions (e.g., correlation within and across matrices, differences in dimensionality between matrices, outliers within and across matrices) they differ. Finally Dray et al. [36] showed the advantages of merging Co-inertia and Procrustean analysis, where the latter is used as a precursor of the former. In reality, future studies are required to contrast Co-inertia and Procrustean analysis, but in either form of analyses we can produce residual vectors (PAM) that can be further analyzed.

Procrustes can be perhaps best justified when the number of predictors is greater than the number of observations or when **X** and **Y** matrices are equally applicable as explanatory and response variables. In plant-soil ecology, for example, above- and below-ground data matrices can be interchanged as explanatory and response variables. Plant community variation has been shown to be related to variation in below-ground compartments [24]. In addition, soil components such as fertility and the microbial community have been proven to influence aspects of vegetation [49]. Thus, with the literature showing that both types of datasets under analysis can structure each other, the use of Procrustes analysis, as a symmetric canonical analysis method, should be encouraged among plant and soil ecologists and ecologists in general. We hope that this paper has provided enough examples of the potential for using the Procrustes framework as a precursor to further explore ecological data.

## Supporting Information

Text S1 **R code showing how to use PAM associated to ordination methods (** **Fig. 4** **in the main text).** For this example we used data from Mitchell et al. [39].
(DOCX)

Text S2 **R code showing how to use the PAM in a Regression framework (** **Fig. 5** **in the main text).**
(DOCX)

Text S3 **R code showing how to use the PAM in an ANOVA framework (** **Fig. 6** **in the main text).**
(DOCX)
