# Supplementary material for: Much beyond Mantel: Bringing Procrustes Association Metric to the Plant and Soil Ecologist’s Toolbox
Source: PLoS One. 2014 Jun 27;9(6):e101238. doi: 10.1371/journal.pone.0101238 (PMC4074130; doi:10.1371/journal.pone.0101238)
Supplement: Text S1 — R code showing how to use PAM associated to ordination methods ( Fig. 4 in the main text). For this example we used data from Mitchell et al. [39]. (DOCX) [file pone.0101238.s001.docx]

**Text S1 R code showing how to use PAM associated to ordination methods (Figure 4 in the main text). For this example we used data from Mitchell et al.[39].**

library(vegan)

library(labdsv)

library(BiodiversityR)

library(mvshapiro.test)

###################################

#######################################

##########################

# Loading files:

#¨¨¨¨¨¨¨¨¨¨¨¨¨¨¨¨¨¨¨¨¨¨¨¨¨¨¨¨¨¨¨¨

bact.m<-read.table(file="BacterialM.txt",header=TRUE,row.names=1)¨

archea.m<-read.table(file="Archea",header=TRUE,row.names=1)

archea.m

fungi.m<-read.table(file="Fungi",header=TRUE,row.names=1)

fungi.m

plfa.m<-read.table(file="PLFA",header=TRUE,row.names=1)

plfa.m

sc.m<-read.table(file="SC",header=TRUE,row.names=1)

sc.m

veg.m<-read.table(file="Vegetation",header=TRUE,row.names=1)

C.bact<-bact.m[1:12,] # TRFLP bacterial at the Craggan site

K.bact<-bact.m[13:24,] #TRFLP bacterial at the Kerrow sites

t.bact<-bact.m[25:35,] # TRFLP bacterial at the Tulchan sites

C.fung<-fungi.m[1:12,] # TRFLP fungal at the Craggan site

K.fung<-fungi.m[13:24,] #TRFLP fungal at the Kerrow sites

t.fung<-fungi.m[25:35,] # TRFLP fungal at the Tulchan site

C.plfa<-plfa.m[1:12,] # PLFA at the Craggan site

K.plfa<-plfa.m[13:24,] #PLFA at the Kerrow sites

t.plfa<-plfa.m[25:35,] # PLFA at the Tulchan site

C.sc<-sc.m[1:12,] # SC at the Craggan site

K.sc<-sc.m[13:24,] # SC at the Kerrow sites

t.sc<-sc.m[25:35,] # SC at the Tulchan site

C.veg<-veg.m[1:12,] # Vegetation at the Craggan site

K.veg<-veg.m[13:24,] # Vegetation at the Kerrow sites

t.veg<-veg.m[25:35,] # Veg at the Tulchan site

#Bacterial.Mittchell.

cbact.bray<-vegdist(C.bact,"bray")

cbact.hel<-decostand(C.bact,"hel")

cbact.chord<-decostand(C.bact,"norm")

cbact.euc<-vegdist(C.bact,"euc")

cbact.jac<-vegdist(C.bact,"jac",binary=TRUE)

cbact.sor<-vegdist(C.bact,"bray",binary=TRUE)

cbact.gower<-distance(C.bact,"gower")

cbact.modgower10<-distance(C.bact,"modgower10")

cbact.modgower2<-distance(C.bact,"modgower2")

cbact.man<-distance(C.bact,"manhattan")

cbact.maha<-distance(C.bact,"mahalanobis")

cbact.canberra<-vegdist(C.bact,"canberra")

cbact.kulc<-vegdist(C.bact,"kulczynski")

cbact.raup<-raupcrick(C.bact)

#######################

#Fungal. Mitchell

cfung.bray<-vegdist(C.fung,"bray")

cfung.hel<-decostand(C.fung,"hel")

cfung.chord<-decostand(C.fung,"norm")

cfung.euc<-vegdist(C.fung,"euc")

cfung.jac<-vegdist(C.fung,"jac",binary=TRUE)

cfung.sor<-vegdist(C.fung,"bray",binary=TRUE)

cfung.gower<-distance(C.fung,"gower")

cfung.modgower10<-distance(C.fung,"modgower10")

cfung.modgower2<-distance(C.fung,"modgower2")

cfung.man<-distance(C.fung,"manhattan")

cfung.maha<-distance(C.fung,"mahalanobis")

cfung.canberra<-vegdist(C.fung,"canberra")

cfung.kulc<-vegdist(C.fung,"kulczynski")

cfung.raup<-raupcrick(C.fung)

#########################

# PLFA

cplfa.bray<-vegdist(C.plfa,"bray")

cplfa.hel<-decostand(C.plfa,"hel")

cplfa.chord<-decostand(C.plfa,"norm")

cplfa.euc<-vegdist(C.plfa,"euc")

cplfa.jac<-vegdist(C.plfa,"jac",binary=TRUE)

cplfa.sor<-vegdist(C.plfa,"bray",binary=TRUE)

cplfa.gower<-distance(C.plfa,"gower")

cplfa.modgower10<-distance(C.plfa,"modgower10")

cplfa.modgower2<-distance(C.plfa,"modgower2")

cplfa.man<-distance(C.plfa,"manhattan")

cplfa.maha<-distance(C.plfa,"mahalanobis")

cplfa.canberra<-vegdist(C.plfa,"canberra")

cplfa.kulc<-vegdist(C.plfa,"kulczynski")

cplfa.raup<-raupcrick(C.plfa)

# soil chemistry

csc.bray<-vegdist(C.sc,"bray")

csc.hel<-decostand(C.sc,"hel")

csc.chord<-decostand(C.sc,"norm")

csc.euc<-vegdist(C.sc,"euc")

csc.jac<-vegdist(C.sc,"jac",binary=TRUE)

csc.sor<-vegdist(C.sc,"bray",binary=TRUE)

csc.gower<-distance(C.sc,"gower")

csc.modgower10<-distance(C.sc,"modgower10")

csc.modgower2<-distance(C.sc,"modgower2")

csc.man<-distance(C.sc,"manhattan")

csc.maha<-distance(C.sc,"mahalanobis")

csc.canberra<-vegdist(C.sc,"canberra")

csc.kulc<-vegdist(C.sc,"kulczynski")

csc.raup<-raupcrick(C.sc)

# vegetation

cveg.bray<-vegdist(C.veg,"bray")

cveg.hel<-decostand(C.veg,"hel")

cveg.chord<-decostand(C.veg,"norm")

cveg.euc<-vegdist(C.veg,"euc")

cveg.jac<-vegdist(C.veg,"jac",binary=TRUE)

cveg.sor<-vegdist(C.veg,"bray",binary=TRUE)

cveg.gower<-distance(C.veg,"gower")

cveg.modgower10<-distance(C.veg,"modgower10")

cveg.modgower2<-distance(C.veg,"modgower2")

cveg.man<-distance(C.veg,"manhattan")

cveg.maha<-distance(C.veg,"mahalanobis")

cveg.canberra<-vegdist(C.veg,"canberra")

cveg.kulc<-vegdist(C.veg,"kulczynski")

cveg.raup<-raupcrick(C.veg)

##########################

#############################

##################################

######################################

############################################

#Creating partial protest function

#**********************************

p.prot<-function(x,y,z){

reg1<-lm(x~z)

reg2<-lm(y~z)

res1<-residuals(reg1)

res2<-residuals(reg2)

pprot<-protest(res1,res2,perm=10000)

yonx<-residuals(pprot)

yx<-as.matrix(yonx)

return(yonx)

}

#Reducing to same dimensionality

#PCA called "the summarized variation of compartments in 3,4,5 PCA axes"

#*************************************************************************

cbact.pca<-rda(cbact.hel, scale=TRUE )

cveg.pca<-rda(cveg.hel, scale=TRUE )

csc.pca<-rda(log(C.sc+1), scale=TRUE )

cfung.pca<-rda(cfung.hel, scale=TRUE )

cplfa.pca<-rda(log(C.plfa+1), scale=TRUE )

# The variation explained by the PCA axes

# Our threshold was > 70 %.

#I have just been used the PCA axes comprising >70% of variation

#***************************************************************8

summary(cbact.pca,scaling=1)

summary(cfung.pca,scaling=1)

summary(cplfa.pca,scaling=1)

summary(cveg.pca,scaling=1)

summary(csc.pca,scaling=1)

# Axes extractions ( summarized variation in 2 PCA axes)

#***********************************************************

cbact.axes<-scores(cbact.pca,display=c("sites"), choice=c(1,2,3,4,5), scaling=1)

cveg.axes<-scores(cveg.pca,display=c("sites"), choice=c(1,2,3,4,5), scaling=1)

csc.axes<-scores(csc.pca,display=c("sites"), choice=c(1,2,3,4,5), scaling=1)

cplfa.axes<-scores(cplfa.pca,display=c("sites"), choice=c(1,2,3,4,5), scaling=1)

cfung.axes<-scores(cfung.pca,display=c("sites"), choice=c(1,2,3,4,5), scaling=1)

# After the scores extractions we need to relate our two PCA matrices

# Let`s carry out the relationship between SC and PLFA community account to Vegetation

# To do this we can to use the function p.prot

#Only Craggan chronosequence

#*****************************

SConPLFA.veg<-p.prot(csc.axes,cplfa.axes,cveg.axes)

SConPLFA.veg # (3 PCA axes; P = 0.033); (4 PCA axes; P = 0.08) (5 PCA axes, P>0.05) (6 PCA axes < 0.05)

VegonPLFA.SC<-p.prot(cveg.axes,cplfa.axes,csc.axes)

VegonPLFA.SC # Is not significant in 3,4,5, but (6 PCA axes; < 0.001)

SConFung.veg<-p.prot(csc.axes,cfung.axes,cveg.axes)

SConFung.veg # Is not significant (6 PCA axes; <0.001)

SConBact.veg<-p.prot(csc.axes,cbact.axes,cveg.axes)

SConBact.veg # ( 3 PCA axes; P = 0.003) (4 PCA axes, P = 0.05) (5 PCA axes, < 0.01) (6 PCA axes; <0.001)

VegonFung.SC<-p.prot(cveg.axes,cfung.axes,csc.axes)

VegonFung.SC # Significant effect (3 PCA axes; P = 0.022) ( 4 PCA axes; P = 0.01) (5 PCA axes; < 0.001) (6 PCA axes; <0.001)

VegonBact.SC<-p.prot(cveg.axes,cbact.axes,csc.axes)

VegonBact.SC # 3 PCA axes; P = 0.186) (5 PCA axes. <0.05) (6 PCA axes; <0.001)

#########################################

#############################################

######################################################

###############################################################

####################################################################

#Reducing to same dimensionality: the Kerrow site solely

#PCA called "the summarized variation of compartments in 2 PCA axes"

#*************************************************************************

kbact.pca<-rda(decostand(K.bact, "hel"), scale=TRUE )

kveg.pca<-rda(decostand(K.veg,"hel"),scale=TRUE )

ksc.pca<-rda(log(K.sc+1), scale=TRUE )

Kfung.pca<-rda(decostand(K.fung,"hel"), scale=TRUE )

kplfa.pca<-rda(log(K.plfa+1), scale=TRUE )

# The variation explained by the PCA axes

# My threshold was > 70 %.

#I have just been used the PCA axes comprising >70% of variation

#***************************************************************8

summary(kbact.pca,scaling=1)

summary(Kfung.pca,scaling=1)

summary(kplfa.pca,scaling=1)

summary(kveg.pca,scaling=1)

summary(ksc.pca,scaling=1)

# Axes extractions (summarized variation in 2 PCA axes)

#***********************************************************

kbact.axes<-scores(kbact.pca,display=c("sites"), choice=c(1,2,3,4), scaling=1)

kveg.axes<-scores(kveg.pca,display=c("sites"), choice=c(1,2,3,4), scaling=1)

ksc.axes<-scores(ksc.pca,display=c("sites"), choice=c(1,2,3,4), scaling=1)

kplfa.axes<-scores(kplfa.pca,display=c("sites"), choice=c(1,2,3,4), scaling=1)

kfung.axes<-scores(Kfung.pca,display=c("sites"), choice=c(1,2,3,4), scaling=1)

# After the scores extractions we need to relate our two PCA matrices

# Let`s carry out the relationship between SC and PLFA community account to Vegetation

# To do this we can to use the function p.prot

#Only Kerrow chronosequence

#*****************************

kSConPLFA.veg<-p.prot(ksc.axes,kplfa.axes,kveg.axes)

kSConPLFA.veg # ( 3 PCA axes; > 0.05) (4 PCA axes, >0.05) (5 PCA axes; > 0.05) (6 PCA axes, <0.01)

kVegonPLFA.SC<-p.prot(kveg.axes,kplfa.axes,ksc.axes)

kVegonPLFA.SC # (3 PCA axes; > 0.05) (4 PCA axes, > 0.05) ( 5 PCA axes; > 0.05) (6 PCA axes, <0,040)

kSConFung.veg<-p.prot(ksc.axes,kfung.axes,kveg.axes)

kSConFung.veg # ( 3 PCA axes; <0.001) (4 PCA axes, < 0.001) (5 PCA axes; <0.001) (6 PCA axes, < 0.001 )

kSConBact.veg<-p.prot(ksc.axes,kbact.axes,kveg.axes)

kSConBact.veg # (3 PCA axes;>0.05) ( 4 PCA axes; < 0.001 ) ( 5 PCA axes; <0.01) ( 6 PCA axes; < 0.001)

kVegonFung.SC<-p.prot(kveg.axes,kfung.axes,ksc.axes)

kVegonFung.SC # (3 PCA axes; <0.001) (4 PCA axes; < 0.001 ) (5 PCA axes; <0.001) ( 6 PCA axes; < 0.001)

kVegonBact.SC<-p.prot(kveg.axes,kbact.axes,ksc.axes)

kVegonBact.SC # (3 PCA axes; > 0.05) ( 4 PCA axes; <0.01) ( 5 PCA axes; < 0.001) ( 6 PCA axes; < 0.001)

########################################

###########################################

##################################################

######################################

#####################################

#Reducing to same dimensionality: the Tulchan chronosequence solely

#PCA called "the summarized variation of compartments in n PCA axes"

#*************************************************************************

tbact.pca<-rda(decostand(t.bact, "hel"), scale=TRUE )

tveg.pca<-rda(decostand(t.veg,"hel"),scale=TRUE )

tsc.pca<-rda(log(t.sc+1),scale=TRUE)

tfung.pca<-rda(decostand(t.fung,"hel"), scale=TRUE )

tplfa.pca<-rda(log(t.plfa+1), scale=TRUE )

# The variation explained by the PCA axes

# My threshold was > 70 %.

# We have just been used the PCA axes comprising >70% of variation

#***************************************************************8

summary(tbact.pca,scaling=1)

summary(tfung.pca,scaling=1)

summary(tplfa.pca,scaling=1)

summary(tveg.pca,scaling=1)

summary(tsc.pca,scaling=1)

# Axes extractions (summarized variation in n PCA axes)

#***********************************************************

tbact.axes<-scores(tbact.pca,display=c("sites"), choice=c(1,2,3,4,5,6), scaling=1)

tveg.axes<-scores(tveg.pca,display=c("sites"), choice=c(1,2,3,4,5,6), scaling=1)

tsc.axes<-scores(tsc.pca,display=c("sites"), choice=c(1,2,3,4,5,6), scaling=1)

tplfa.axes<-scores(tplfa.pca,display=c("sites"), choice=c(1,2,3,4,5,6), scaling=1)

tfung.axes<-scores(tfung.pca,display=c("sites"), choice=c(1,2,3,4,5,6), scaling=1)

# After the scores extractions we need to relate our two PCA matrices

# Let`s gconduct the relationship between SC and PLFA community account to Vegetation

# To do this we can to use the function p.prot

#Only Tulchan chronosequence

#*****************************

tSConPLFA.veg<-p.prot(tsc.axes,tplfa.axes,tveg.axes)

tSConPLFA.veg # ( 3 PCA axes; > 0.05) 4 PCA axes > 0.05 (5 PCA axes; < 0.05)

# 6PCA axes < 0.001

tVegonPLFA.SC<-p.prot(tveg.axes,tplfa.axes,tsc.axes)

tVegonPLFA.SC # (3 PCA axes; < 0.01) 4 (PCA axes < 0.01) ( 5 PCA axes < 0.001)

# 6PCA axes, <0.001

tSConFung.veg<-p.prot(tsc.axes,tfung.axes,tveg.axes)

tSConFung.veg # ( 3 PCA axes; > 0.05) 4 PCA axes > 0.05; (5 PCA axes; <0.01)

# 6 PCA axes, <0.001

tSConBact.veg<-p.prot(tsc.axes,tbact.axes,tveg.axes)

tSConBact.veg # (3 PCA axes;<0.01) 4 PCA axes < 0.01 (5PCA axes, < 0.001)

# 6 PCA axes; <0.001

tVegonFung.SC<-p.prot(tveg.axes,tfung.axes,tsc.axes)

tVegonFung.SC # (3 PCA axes; >0.05) 4 PCA axes < 0.05; (5 PCA axes < 0.001)

#6 PCA axes; <0.001

tVegonBact.SC<-p.prot(tveg.axes,tbact.axes,tsc.axes)

tVegonBact.SC # (3 PCA axes; < 0.05); 5 PCA axes; < 0.001

# 6 PCA axes; < 0.001

p.prot<-function(x,y,z){

reg1<-lm(x~z)

reg2<-lm(y~z)

res1<-residuals(reg1)

res2<-residuals(reg2)

pprot<-protest(res1,res2,perm=10000)

yonx<-residuals(pprot)

yx<-as.matrix(yonx)

return(yonx)

}

##############################

# All of Procrustean association metric derived

# from 6 PCA axes were used to build a matrix of "effects/relationship within each chronosequence

#Each line of dataframe is an "effect" of Soil chemistry and Plant community on the overall microbial community (PLFA),

# Bacterial (T-RFLP) and Fungal (T-RFLP) from a Procrustes relationship based on 6 PCA axes matrices.

ordcra<-read.table(file="OrdCra.txt",header=TRUE,row.names=1)

ordker<-read.table(file="OrdKer.txt",header=TRUE,row.names=1)

ordTul<-read.table(file="OrdTu.txt",header=TRUE,row.names=1)

#Ordinating PAM matrices from each chronosequence:

pca.ordker<-rda(ordker)

ordiplot(pca.ordker,type="t",display=c("sites"),choice=c(1,2),scaling=1)

summary(pca.ordker,scaling=1)

pca.ordtul<-rda(ordTul)

ordiplot(pca.ordtul,type="t",display=c("sites"),choice=c(1,2),scaling=1)

summary(pca.ordker,scaling=1)

pca.ordcra<-rda(ordcra)

ordiplot(pca.ordcra,type="t",display=c("sites"),choice=c(1,2),scaling=1)

summary(pca.ordcra,scaling=1)

######

#Creating Graphs of Procrustes

ordiplot(pca.ordcra,type="t",display=c("sites"),choice=c(1,2),scaling=1)

ordiplot(pca.ordker,type="t",display=c("sites"),choice=c(1,2),scaling=1)

ordiplot(pca.ordtul,type="t",display=c("sites"),choice=c(1,2),scaling=1)
