# Supplementary material for: Much beyond Mantel: Bringing Procrustes Association Metric to the Plant and Soil Ecologist’s Toolbox
Source: PLoS One. 2014 Jun 27;9(6):e101238. doi: 10.1371/journal.pone.0101238 (PMC4074130; doi:10.1371/journal.pone.0101238)
Supplement: Text S2 — R code showing how to use the PAM in a Regression framework ( Fig. 5 in the main text). (DOCX) [file pone.0101238.s002.docx]

**Text S2 R code showing how to use the PAM in a Regression framework (Figure 5 in the main text).**

##

# Hypothetical example

#Procrustes analysis associated with ANOVA

# Hypothetical datasets

#******************************

# Soil Microbial structure raw data [X] PLFA data set

# Soil microbial functioning raw data [Y] ( denitrifiers (nirS, nirK, nosZ), nitrifiers (amoA), methanotrophic gene (pmoA) )

# Land use types (LU) factor with four levels: Original forest fragment, Silvipastoral system, improved pasture, unimproved pasture

###################################################################

###################################################################

######################################################################

# Running the principal components analyses and making PC matrices from [X] and [Y]

#*************************************************************

# load vegan R package:

library(vegan)

# PCA of [X] (PLFA data set):

#*****************************

X.log<- log(X+1) # transformation

X.pca<- rda(X.log)

# Extracting and obtaining principal components of X

X.2axes<- scores(X.pca, display = c("sites"), choice = c(1,2)) # 2 PCA axes

X.2axes<-as.matrix(X.2axes) # 2 axes matrix

# PCA of [Y] ( denitrifiers (nirS, nirK, nosZ), nitrifiers (amoA), methanotrophic gene (pmoA) ))

#********************************

Y.log<- log(Y+1)

Y.pca <-rda(Y.log)

# Extracting and obtaining principal components matrices of Y

Y.2axes<- scores(Y.pca, display = c("sites"), choice = c(1,2)) # 2 PCA axes

Y.2axes<-as.matrix(Y.2axes) # 2 axes matrix

#####################################

####################################

####################################

########################################

##########################################

# Run the Procrustes relationships between X and Y axes matrices

#***************************************************************

# Between 2 axes matrices (Figure 6a. Main text)

bet2axes<-Procrustes(X.2axes,Y.2axes) # Notice both matrices must be the same number of columns

#######################################################

########################################################

########################################################

# Obtaining the PAMs (Procrustes association metric)

PAM2axes<-residuals(bet2axes) # Relationship between SMC-SMF based on 2 axes matrices

######################################

############################################

################################################

# Run an ANOVA with Land use (LU) as fixed factor and Soil microbial structure-soil microbial functioning, i.e PAM2axes as response

#********************************************************************************************************************************

datafr<-data.frame(cbind(PAM2axes,LU)

ANOVA.proc<-aov(PAM2axes~LU,datafr)

ANOVA.proc # In our hypothetical example, the one-way ANOVA output shows that at least two types of land

#use differ statistically (F = 7.047, P = 0.003)

#########################################################3

################################

###################################

##################################

# Running a multiple comparisons mean test to assess different land use types in regard to their effect

# on the relationship between soil microbial structure and functioning.

#****************************************************************************

#load agricolae R package

HSD.test(ANOVA.proc,"LU",group=TRUE) # Tukey 95%

#Finish
