# Supplementary material for: Much beyond Mantel: Bringing Procrustes Association Metric to the Plant and Soil Ecologist’s Toolbox
Source: PLoS One. 2014 Jun 27;9(6):e101238. doi: 10.1371/journal.pone.0101238 (PMC4074130; doi:10.1371/journal.pone.0101238)
Supplement: Text S3 — R code showing how to use the PAM in an ANOVA framework ( Fig. 6 in the main text). (DOCX) [file pone.0101238.s003.docx]

**Text S3 R code showing how to use the PAM in an ANOVA framework (Figure 6 in the main text).**

# Hypotethical example

#Procrustes analysis associated with variation partitioning

# Hypothetical datasets

# Soil Microbial Community raw data [X]

# Soil microbial functioning raw data [Y]

# Soil properties [SP]

# Spatial information raw data [SIrd]

################################################

#################################################

###############################################3

# Obtaining the PCNM matrix from a equispaced grid ( 10 x 10 ). Here we are following

http://www.ualberta.ca/~ahamann/teaching/renr690/Lab9.pdf

# 1: To load PCNM package:

library(PCNM)

# 2: obtaining dist matrix from [SIrd]:

SIrd.dist.hypo <- dist(SIrd)

# 3: Obtaining PCNM functions automatically by using PCNM function:

pcnm.auto.hypo <- PCNM(SIrd)

# 4: finding the truncation distance by plotting the minimum spanning tree:

spantree(pcnm.auto,hypo$spanning, SIrd)

threshold.hypo <- pcnm.auto.hypo$thresh #truncation distance (Minimum distance connecting all sampling points)

#In our hypotethical example, the truncated distance was defined as 1 m.

# 5: Moran index for each PCNM variables that has been found out.

pcnm.auto.hypo$Moran_I

# 6: PCNM variables having Moran index higher than Moran'I expected, i.e, with positive spatial correlation

pos.pcnm <- which(pcnm.auto.hypo$Moran_I$Positive == T) # In our hypotethical example, 28 PCNMs variables were selected as describing a positive spatial correlation

# 7: making a positive PCNM dataframe:

data.frame(pcnm.auto.hypo$vectors)[,pos.pcnm] # This dataframe gathering together all PCNM variables accounting to a psoitive spatial correlation

# This dataframe will be used as predictor in a variance partitioning

##########################################

#########################################

# Running the principal components analyses and making PC matrices from [X] and [Y]

#*************************************************************

# load vegan R package:

library(vegan)

# PCA of [X] (PLFA data set):

#*****************************

X.log<- log(X+1) # transformation

X.pca <- rda(X.log)

# Extracting and obtaining principal components of X

X.2axes<- scores(X.pca, display = c("sites"), choice = c(1,2)) # 2 PCA axes

X.2axes<-as.matrix(X.2axes) # 2 axes matrix

X.3axes<- scores(X.pca, display = c("sites"), choice = c(1,2,3)) # 3 PCA axes

X.3axes<-as.matrix(X.3axes) # 3 axes matrix

X.naxes<- scores(X.pca, display = c("sites"), choice = c(1,2,3,...,n)) # n PCA axes

X.naxes<-as.matrix(X.naxes) # n axes matrix

# PCA of [Y] (Enzyme activities)

#********************************

Y.log<- log(Y+1)

Y.pca <-rda(Y.log)

# Extracting and obtaining principal components matrices of Y

Y.2axes<- scores(Y.pca, display = c("sites"), choice = c(1,2)) # 2 PCA axes

Y.2axes<-as.matrix(Y.2axes) # 2 axes matrix

Y.3axes<- scores(Y.pca, display = c("sites"), choice = c(1,2,3)) # 3 PCA axes

Y.3axes<-as.matrix(Y.3axes) # 3 axes matrix

Y.naxes<- scores(Y.pca, display = c("sites"), choice = c(1,2,3,...,n)) # n PCA axes

Y.naxes<-as.matrix(Y.naxes) # n axes matrix

#####################################

####################################

####################################

########################################

##########################################

# Run the Procrustes relationships between X and Y axes matrices

#***************************************************************

# Between 2, 3 and n axes matrices

bet2axes<-procrustes(X.2axes,Y.2axes) # Notice both matrices must be the same number of columms

bet3axes<-procrustes(X.3axes,Y.3axes)

betnaxes<-procrustes(X.naxes,Y.naxes)

#############################################

###########################################

######################################

# Obtaining the PAMs (Procrustes association metric)

PAM2axes<-residuals(bet2axes) # Relationship between SMC-SMF based on 2 axes matrices

PAM3axes<-residuals(bet3axes)

PAMnaxes<-residuals(betnaxes)

####################################

#################################

# Run variation partitioning

#*******************************

# Responses: PAM2axes, PAM3axes and PAMnaxes

# Predictors: pos.pcanm (neutral processes) and soil properties (niche processes)

# SP.log<-log(SP+1) # logtransformation of soil properties matrix

proc.varp2axes<-varpart(PAM2axes,SP.log,pos.pcnm)

proc.varp2axes # In the output we will have the fractions [a], [b] and [c] depicting the individual contribution of

# niche processes [a]; neutral processes [c] and both [c] on

#the soil microbial community-soil microbial functioning relationship (PAM2axes)

# In our hypothetical example we generated a Venn diagram to depicting the contribution of

# neutral and niche process. In this example the contribution of neutral processes (unmeasured spatial factors)

# on the SMC-SMF relationship was lower than niche process effect.
